# Xiaoyao San alleviates emotional distress - induced TNBC growth through augmenting intratumoral CD8^+^T cell infiltration mediated by Rela/NF-kB-Cxcl9 axis

**DOI:** 10.3389/fimmu.2026.1762492

**Published:** 2026-02-05

**Authors:** Yuqi Liang, Yingchao Wu, Jieting Chen, Huan Shi, Zhuang Li, Juexiao Zeng, Junfeng Huang, Qian Zuo, Lingling Ye, Xue Song, Yan Dai, Yunlong Bai, Qianjun Chen

**Affiliations:** 1 Department of Breast, Guangdong Provincial Hospital of Chinese Medicine, The Second Affiliated Hospital of Guangzhou University of Chinese Medicine, The Second Clinical College of Guangzhou University of Chinese Medicine, State Key Laboratory of Traditional Chinese Medicine Syndrome, Guangzhou, Guangdong, China; 2 Chinese Medicine Guangdong Laboratory, Hengqin, Guangdong, China; 3 Key Laboratory of Chinese Medicinal Resource from Lingnan, Ministry of Education, School of Pharmaceutical Sciences, Guangzhou University of Chinese Medicine, Guangzhou, Guangdong, China

**Keywords:** CD8^+^T cell, Cxcl9, emotional distress, triple-negative breast cancer, Xiaoyao San

## Abstract

**Background:**

Emotional distress (ED) is closely associated with the progression of triple-negative breast cancer (TNBC). Xiaoyao San (XYS), a classical Chinese herbal prescription traditionally used for mood regulation, has demonstrated potential therapeutic efficacy in emotion-related breast cancer. However, the molecular mechanisms through which XYS mitigates ED-induced TNBC (ED-TNBC) remain insufficiently characterized. This study aimed to investigate the therapeutic effects of XYS on ED-TNBC and elucidate its underlying molecular mechanisms.

**Methods:**

A TNBC mouse model subjected to chronic unpredictable mild stress (CUMS) was developed to simulate ED-TNBC conditions. The therapeutic efficacy of XYS at varying doses was evaluated through behavioral assessments and tumor growth analyses. Multi-omics analyses integrating network pharmacology, bioinformatics, and molecular dynamics simulations were employed to identify principal active constituents and key molecular targets of XYS. Transcriptomic profiling, *in vivo* and *in vitro* functional assays, and molecular biology experiments were conducted to delineate the molecular mechanisms underlying XYS-mediated regulation.

**Results:**

High-dose XYS markedly alleviated depression-like behaviors and suppressed ED-TNBC tumor progression, with no evident adverse effects observed. Transcriptomic and molecular analyses revealed that XYS enhanced CD8^+^ T cell infiltration and cytotoxic activity through activation of Cxcl9. The active constituents of XYS were found to bind directly to the transcription factor Rela. Subsequent experiments verified that Cxcl9 secretion from TNBC cells depends on Rela activity. In addition, XYS upregulated Rela expression and promoted its nuclear translocation.

**Conclusion:**

XYS directly targets and activates the intratumoral Rela/NF-κB–Cxcl9 axis, promoting CD8^+^T cell infiltration and activation, thereby inhibiting the growth of ED-TNBS.

## 1 Introduction

Breast cancer (BC) remains the most common malignancy and a principal cause of cancer-related mortality among women globally. In 2022, approximately 2.3 million new cases and 670,000 deaths were documented, representing 25% of all female cancers and 15.5% of cancer-related deaths (1). Triple-negative BC (TNBC), accounting for 15–20% of BC cases, is defined by highly aggressive biological behavior, unfavorable prognosis, and limited therapeutic options (2, 3). Consequently, despite extensive research, identifying effective treatments for TNBC remains an elusive goal. In recent years, oncology research has transitioned from a focus on intrinsic genetic alterations within tumor cells to the exploration of extrinsic host-related influences. Emotional distress (ED), particularly depression, has been identified as a factor that accelerates TNBC progression and diminishes treatment efficacy (4). Preclinical evidence indicates that selective serotonin reuptake inhibitors (SSRIs) partially inhibit ED-induced TNBC growth (ED-TNBC) (5); however, a cohort study data reveal a strong correlation between SSRI administration and increased BC mortality risk (6), highlighting an urgent requirement for safer and more effective therapeutic strategies.

An immunosuppressive tumor microenvironment (TME) constitutes a important feature contributing to the initiation and progression of ED-driven malignancies. Among immune effector populations, CD8^+^ T cells serve as the principal cytotoxic subset within the TME, directly eradicating tumor cells and sustaining immune surveillance (7, 8). ED markedly diminishes CD8^+^ T cell infiltration and cytotoxic competence, thereby attenuating antitumor immune responses (9, 10). The recruitment of CD8^+^ T cells into tumor tissue depends on specific chemokines (11, 12). Among them, C-X-C motif chemokine ligands 9 (Cxcl9) exhibits the strongest association with CD8^+^ T cell density and antitumor activity in solid tumors and plays a central role in sustaining their cytolytic function (13, 14). Regulation of Cxcl9 expression primarily occurs through interferon-γ (IFN-γ) signaling and the NF-κB (Rela) pathway (15, 16), indicating that modulation of the Cxcl9 axis may counteract ED-induced immunosuppression within the TME and provide a promising therapeutic avenue for ED-TNBC.

Traditional Chinese medicine (TCM), characterized by a holistic therapeutic philosophy and a favorable safety profile, has gained increasing attention as an adjunctive approach in cancer treatment (17). Xiaoyao San (XYS), a classical formula first documented in *Taiping Huimin Heji Ju Fang* during the Song Dynasty, has been extensively applied in managing liver Qi stagnation, gynecological disorders, and ED-associated conditions. It is well recognized for its antidepressant efficacy and potential anticancer activity (18, 19). The prescription comprises eight herbal components: *Angelica sinensis* (Oliv.) Diels (Angelicae Sinensis Radix, ASR), *Atractylodes macrocephala Koidz.* (Atractylodis Macrocephalae Rhizoma, AMR), *Bupleurum chinese DC* (Bupleuri Radix, BR), *Poria cocos* (Schw.) Wolf. (PW), *Paeonia lactiflora Pal*l (Paeoniae Radix Alba, PRA), *Glycyrrhiza uralensis* Fisch. (Glycyrrhizae Radix et Rhizoma, GRR), *Mentha canadensis Lamiaceae* (Herba Menthae, HM), and *Zingiber officinale Roscoe* (Zingiberis Recens, ZR) (19). A growing body of clinical and systematic evidence indicates that XYS not only improves emotional well-being and mitigates depressive symptoms but also enhances therapeutic outcomes in cancer treatment. Owing to its dual regulatory effects on mood and tumor biology, XYS has become one of the most frequently prescribed formulas for BC patients, particularly those experiencing concomitant ED (20–22). However, the molecular basis underlying the modulatory effects of XYS on ED-associated TNBC remains insufficiently elucidated.

This study integrated computational pharmacology, transcriptomic profiling, and *in vivo/in vitro* validation to evaluate the therapeutic potential of XYS in ED-TNBC. XYS treatment alleviated depression-like behaviors and suppressed ED-TNBC progression by enhancing CD8^+^ T cell infiltration and cytotoxic activity. Through systematic analysis of therapeutic pathways, major chemical constituents, and their molecular targets, the mechanism of XYS was identified to involve modulation of the Rela/NF-κB–Cxcl9 axis. Collectively, the results clarify the anti-TNBC efficacy and mechanistic basis of XYS under ED conditions and highlight the therapeutic promise of targeting the Cxcl9 or Rela/NF-κB pathway for managing ED-cancers.

## 2 Materials and methods

### 2.1 Preparation of XYS

XYS were procured from Beijing Boran Pharma Co. Ltd. (Beijing, China, production batch number 240401). This traditional formulation incorporates eight primary botanical ingredients (**Table 1**). Verification of taxonomic nomenclature was performed through the Medicinal Plant Names Services database (MPNS; https://mpns.science.kew.org/). Quality control procedures adhered to the 2025 Chinese Pharmacopoeia guidelines, utilizing paeoniflorin as the analytical standard.

**Table 1:** Composition of Xiaoyao San (XYS).

Chinese name	Latin names	Plant part	Dose (g)
Chai Hu	*Bupleurum chinense DC.*	Roots	143
Dang Gui	*Angelica sinensis (Oliv.) Diels.*	Roots	143
Bai Shao	*Paeonia lactiflora Pall.*	Roots	143
Bai Zhu	*Atractylodes macrocephala Koidz.*	Roots	143
Fu Ling	*Smilax glabra Roxb.*	Seeds	143
Sheng Jiang	*Zingiber officinale Roscoe*	Roots	143
Gan Cao	*Glycyrrhiza glabra l.*	Roots	114.4
Bo He	*Mentha arvensis l.*	Aerial parts	28.6

### 2.2 UPLC-Q-Orbitrap-MS analysis

The phytochemical analysis of XYS involved processing 0.1 g samples through a standardized protocol. Initial extraction combined the powdered material with 1 mL 70% methanol and grinding beads for mechanical disruption (3 min), supplemented by cold ultrasonic treatment (10 min). After 4°C centrifugation, the clarified extract was membrane-filtered for LC-MS analysis. Chromatography was executed with Zorbax Eclipse C18 column (100 mm × 2.1 mm, 1.8 μm) at 30°C with 0.3 mL/min flow, employing a gradient of 0.1% formic acid/water (A) and methanol (B) as detailed in **Table 2**. The Q-Orbitrap mass spectrometry (UPLC-Q-Orbitrap-MS) incorporated full-scan acquisition (100–1500 m/z, 120K resolution) and data-dependent MS/MS (Top10 HCD, 60K resolution) on a Q-Orbitrap platform. Final compound characterization was accomplished through mzCloud database matching using Compound Discoverer 3.3.

**Table 2:** LC mobile phase conditions.

Time (min)	Flow rate (μL/min)	Gradient	B % Acetonitrile	A % Fomic acid
0-2	300	–	5	95
2-6		Linear gradient	30	70
6-7		–	30	70
7-12		Linear gradient	78	22
12-14		–	78	22
14-17		Linear gradient	95	5
17-20		–	95	5
20-21		Linear gradient	5	95
21-25		–	5	95

### 2.3 Cell lines and culture

Zhejiang Meisen Cell Technology (Hangzhou) provided the Py230 TNBC cell line, which was grown in standard culture conditions (37°C, 5% CO_2_) using medium containing 10% heat-inactivated FBS and 1% penicillin-streptomycin antibiotic mixture.

### 2.4 Lentivirus establishment and transfection

To modulate gene expression, Py230 cells underwent transfection using lentiviral constructs sourced from TranSheep Bio (Shanghai, China). These vectors were engineered either to induce *Cxcl9* and *Rela* overexpression or to suppress *Cxcl9* and *Rela* expression via specific short hairpin RNAs (shRNAs). A corresponding non-targeting shRNA was utilized as a negative control. At 48 hours post-transfection, the effectiveness of the genetic modifications was verified by Western blot analysis. The specific sequences for all lentiviral constructs used are provided in **Table 3**.

**Table 3:** The sequence of lentiviruses.

NO.	Target sequences
Control-shRNA	TTCTCCGAACGTGTCACGT
*Cxcl9*-shRNA	TCGTCGTTCAAGGAAGACTA
*Rela*-shRNA	GGACCTATGAGACCTTCAAGA

### 2.5 Animals model

Experiments utilized 6-8-week-old female C57BL/6J mice (18–20 g; Beijing Weitong Lihua) housed in pathogen-free conditions (25 ± 2°C, 50 ± 10% humidity, 12 h light cycle) with unrestricted access to food/water. The Institutional Animal Care Committee of Guangdong Province Hospital of Chinese Medicine approved all protocols (2022079), conducted per National Institutes of Health’s guidelines (NIH Publication No. 80-23, revised in 1996).

Chronic ED was induced via 4-week chronic unpredictable mild stress (CUMS) paradigm with daily random stressors (food/water restriction, 45° tilted cage, restraint, moist bedding and so on). After orthotopic Py230 cell inoculation (1×10^6^ cells/100 μL PBS), seven experimental groups (n=6) were established: control (Ctrl), low-dose XYS (XYS^low^, 3.25 g/kg), high-dose XYS (XYS^high^, 6.5 g/kg), CUMS, CUMS+ XYS^low^, CUMS+ XYS^high^, and CUMS+ Fluoxetine (FLX, 20 mg/kg, Lilly Co., Ltd., Suzhou, Jiangsu, China). Treatments were administered via gavage for 6 weeks, with controls receiving saline.

To examine Cxcl9 involvement, supplementary groups (n=6) comprised: CUMS, CUMS+hCxcl9, CUMS+XYS (6.5 g/kg), and CUMS+XYS+anti-Cxcl9 (BE0309 mAb, 200 μg/10μl intratumoral q2d). Isotype controls received Armenian hamster IgG (BioXCell BP0091, USA) intraperitoneally.

### 2.6 Behavioral tests

#### 2.6.1 Sucrose preference test

Rodents were acclimated to 1% sucrose solution prior to testing. Following a 24-hour period of deprivation, the animals were presented concurrently with two pre-weighed bottles containing sucrose solution and pure water, respectively. Bottle positions were rotated at 6 h intervals to prevent side preference. Fluid intake was measured gravimetrically after 12 h, with the sucrose preference quantified as: (sucrose intake/total liquid consumption) ×100%.

#### 2.6.2 Tail suspension test

Animals were subjected to tail suspension in a dedicated chamber (55×60×11.5 cm) for 300 sec. Behavioral despair was objectively quantified as cumulative immobility time using the SMART video tracking platform (V.3.0, Panlab).

#### 2.6.3 Open field test

Exploratory behavior was examined in a 50 cm^3^ arena under low-light conditions. Individual mice were observed for 5 min, with movement trajectories recorded by infrared videography and analyzed via SMART software (V.3.0, Panlab) to evaluate ambulatory patterns.

### 2.7 Immunohistochemistry and TUNEL analysis

For histopathological analysis, excised tumors were immersed in 4% PFA (24 h, 4°C) and subsequently paraffin-embedded. Microtome sections (4 μm) were transferred to adhesive slides and heat-fixed. Prior to staining, sections underwent xylene clearing (2×10 min) and graded alcohol rehydration. Immunostaining followed established methods (23) with antibodies specific for Bcl-2, Bax, cleaved-caspase-3, Ki67, and Rela/p-Rela at indicated dilutions. TUNEL staining required proteinase K pretreatment (20 μg/mL, 37°C, 15 min) before fluorescent labeling (Roche TUNEL reagent, 60 min, 37°C). Sections were viewed on Olympus BX51 microscope after its nuclei were identified by DAPI.

### 2.8 RNA sequencing

Total RNA was isolated from excised murine tumors and quality-controlled using Agilent’s 2100 Bioanalyzer. Library preparation commenced with messenger RNA (mRNA) capture via poly-A selection beads, followed by cDNA synthesis incorporating end repair and A-tailing steps. Adapter-ligated fragments (370–420 bp) were size-selected before PCR amplification. Sequencing utilized the Illumina NovaSeq 6000 platform. instrument. Initial data processing involved adapter trimming and quality filtering (fastp). Genome-aligned reads were quantified (FeatureCounts v1.5.0-p3) prior to multivariate analysis (principal component analysis (PCA), OmicShare Tools). DESeq2 (v1.20.0) identified statistically significant differentially expressed genes (DEGs) (padj<0.05, |log2FC|≥1.3). We utilized the Xiantao academic platform (http://www.xiantao.love) and the STRING database (https://cn.string-db.org) (24) to conduct enrichment and immune infiltration analyses, as well as to construct protein-protein interaction (PPI) networks.

### 2.9 CCK-8 cell viability assay

The determination of cell viability was performed via the CCK-8 kit (C0037, Beyotime, China) as per the protocols reported in our previous work (23).

### 2.10 Flow cytometry analysis

Single-cell preparations were obtained from tumor specimens using a standardized enzymatic digestion procedure (25). After tissue mincing, samples were incubated with collagenase I (1 mg/mL) and DNase I (0.2 mg/mL) in RPMI 1640 at physiological temperature for 4 hours. Cellular debris was removed by filtration through a 70 μm strainer, followed by red blood cell lysis and PBS washing prior to staining. For immunophenotyping, anti-CD45-APC-CY7 (557659, BD Biosciences), anti-CD3-FITC (100204, Biolegend), anti-CD8-APC (553035, BD Biosciences), anti-CD16/CD32 (553142, BD Biosciences) and Fixable Viability Stain 450 (562247, BD Biosciences) were stained. Intracellular targets were detected post-permeabilization: anti-Granzyme B (GZMB)-Percp/Cyanine5.5 (372212, BD Biosciences), anti-TNF-PE (554419, BD Biosciences) or anti-TNF-PE/Cyanine7 (506324, Biolegend). All reagents were used at 1:100 dilution before flow analysis. In the flow cytometric analysis of tumor tissues, for each sample, 1 × 10^6^ events were gathered, with 6 biological replicates per group. For the flow cytometric assessment of CD8^+^T cell migration, all cells from each well were collected and analyzed, with three replicates per group. In the analysis of CD8^+^T cell function, 1×10^5^ events were collected per sample, with 3 replicates per group. For flow cytometry analysis, cells were first gated based on forward and side scatter to exclude debris, followed by doublet exclusion. Dead cells were excluded using Fixable Viability Stain 450. CD8^+^ T cells were identified by sequential gating on CD45, CD3, and CD8. The expression of GZMB and TNF-α was analyzed within the gated CD8^+^ T cell population.

### 2.11 ELISA

Quantification of chemokines (Cxcl9, Cxcl10, Cxcl11, Ccl5) in tumor tissues or cell supernatant were conducted using commercially available ELISA kits (Huamei Biological Engineering Co., Wuhan, China) per the manufacturer’s guidelines. After measuring absorbance at 450 nm, protein concentrations were derived from standard curves.

### 2.12 Quantitative real-time polymerase chain reaction

We utilized TRIzol reagent (Takara, China) to extract total RNA, which was then subjected to amplification using an Applied Biosystems PCR platform. The primers used for this assay were designed by Sangon Biotech (Shanghai, China), and their sequences are summarized in **Table 4**.

**Table 4:** Primer sequences.

Gene	Forward (5’-3’)	Reverse (5’-3’)
*Actin*	CTGTGGCATCCATGAAACTACA	GTAATCTCCTTCTGCATCCTGTCA
*Cxcl9*	GTGTGGAGTTCGAGGAACCCT	GGCAGGTTTGATCTCCGTTCT

### 2.13 Western blotting

Protein samples were prepared according to established methods (26). In brief, tumor or cellular proteins were extracted with RIPA buffer and quantified (BCA assay, P0010S; Beyotime, Shanghai, China). After SDS-PAGE separation and transfer to PVDF membranes (GVHP04700; Merck, Darmstadt, Germany), blots were blocked and probed with specific antibodies: anti-Cxcl9 (1:1000, MABS2239, Sigma-Aldrich), anti-Histone3 (1:2000, 17168-1-AP, Proteintech), anti-Rela (1:1000, F0006, Selleck), anti-p-Rela (1:1000, F0155, Selleck), and anti-Actin (1:1000, 67121-1-Ig, Proteintech). HRP-conjugated secondary antibodies (1:3000) were applied before ECL detection (P0018S; Beyotime) and digital imaging (ChemiDox™; Bio-Rad, USA). Quantitative analysis employed ImageJ software with normalization to loading controls. Each western blot was repeated at least 3 times.

### 2.14 Transwell assays

Transwell migration assays (5 μm pores, FTW064, Beyotime) were employed to evaluate CD8^+^T cell chemotaxis. The lower chamber contained 1×10^5^ tumor cells comprising wild-type Py230 cells and their genetically engineered derivatives (*Cxcl9/Rela* -overexpressing or *Cxcl9/Rela*-knockdown variants). In inhibitor studies, tumor cells were pre-treated with either anti-Cxcl9 mAb (BE0309 mAb, 100 ng/mL) or JP-163-16 (HY-174864, 0.2 μM). Isolated splenic CD8^+^T cell (STEMCELL 19853) were stimulated with CD3/CD28 (5 μg/mL) and IL-2 (10 ng/mL), then added to upper chambers (3×10^5^ cells/well). Following 48 h co-culture, transmigrated CD8^+^T cell was counted via flow cytometry.

### 2.15 Network pharmacology

Bioactive compounds from UPLC-Q-Orbitrap-MS profiling were first selected via TCMSP database (https://old.tcmsp-e.com/tcmsp.php) based on pharmacokinetic standaed (OB>30%, DL>0.18) (27). Corresponding protein targets were retrieved from multiple sources (TCMSP, TCMIP (https://www.tcmip.cn), TCM-Suite (http://tcm-suite.aimicrobiome.cn)) and standardization of protein identifiers was used UniProt (https://www.uniprot.org/). Intersection analysis with transcriptomic DEGs identified core targets, which were then analyzed for interactions using STRING (https://cn.string-db.org/, *Mus musculus*, confidence > 0.7) (24). Network topology analysis (CytoHubba Maximal Clique Centrality (MCC) algorithm) revealed hub genes, whose biological functions were characterized through pathway enrichment (Xiantao academic platform, www.xiantao.love).

### 2.16 Cellular thermal shift assay

Cellular disruption of Py230 cells was achieved through triple freeze-thaw in liquid nitrogen. After division into control and treatment groups (10% XYS serum, 30 min, RT), lysates were exposed to varying thermal conditions (49-70°C) with subsequent ice quenching. Western blot analysis detected temperature-dependent protein profiles. Each test was repeated at least 3 times.

### 2.17 Molecular docking

The macromolecular structure of Rela (PDB: 1LE9) from the Protein Data Bank (PDB, https://www.rcsb.org/) was selected as the target protein and prepared using the Protein Preparation Wizard (Schrödinger 2022-1) for dehydration, protonation, and restraint minimization. The binding pocket was defined based on the known active site coordinates. For ligand preparation, compounds from PubChem (https://pubchem.ncbi.nlm.nih.gov/) were processed with the LigPrep module to generate 3D conformers (max 32 stereoisomers) and optimized under the OPLS3e force field at pH 7.0+/-2.0. Docking simulations were carried out using the Standard Precision (SP) protocol within the Schrödinger Ligand Docking module. Finally, the interaction modes were visualized and analyzed using Accelrys Discovery Studio 3.5 (Accelrys, Inc., USA) (28).

### 2.18 Molecular dynamics simulation

Protein-ligand binding dynamics were assessed through molecular dynamics simulations implemented in Gromacs 2023.3 (29). The workflow comprised: (1) System preparation via CHARMM-GUI employing CHARMM36 parameters (30, 31), with solvation in a 10 Å TIP3P water box containing physiological ion concentrations (32, 33); (2) Energy optimization using steepest descent algorithm (convergence criterion: Fmax <1000 kJ·mol^-^¹·nm^-^¹); (3) Stepwise equilibration including NVT (50 ps, 310 K) and NPT (100 ps, 1 bar) phases; (4) Unrestrained 100 ns NPT production run. Trajectory analysis evaluated complex stability through RMSD, RMSF, radius of gyration, and hydrogen bonding metrics.

### 2.19 Luciferase reporter assays

Prediction of specific Rela interaction sites upstream of the Cxcl9 gene was conducted using the JASPAR online tool (34). For the assay, HEK293T cells were plated at a density of 1 × 10^4^ cells/mL in 24-well dishes. Co-transfection was carried out using PolyJet reagent, delivering the *Cxcl9* promoter sequence (cloned into pGL3 Basic) and the Rela expression plasmid (pCR3.1). To normalize for transfection efficiency, the pTK Renilla vector (Promega) was included in all wells. Luciferase signals were quantified 24 hours post-transfection using the Dual-Luciferase^®^ Reporter Assay System (Promega). Luminescence was recorded on a Spark multimode reader (Tecan), and data were calculated as the ratio of Firefly to Renilla activity.

### 2.20 Statistical analysis

Statistical processing and visualization were performed using SPSS 26.0 and GraphPad Prism 9. Results were presented as mean ± SD. Depending on the normality and variance of the data, group differences were analyzed using independent t-tests, Mann–Whitney U-tests, or ANOVA (one-way or Welch’s). *Post hoc* comparisons included Tukey’s test, Dunnett’s T3 test, or the Dunn-Bonferroni method where applicable. Detailed methodology is described in our previously published article (23).

## 3 Results

### 3.1 XYS inhibited the depression-like behaviors and TNBC growth induced by ED *in vivo*

**Figure 1:**
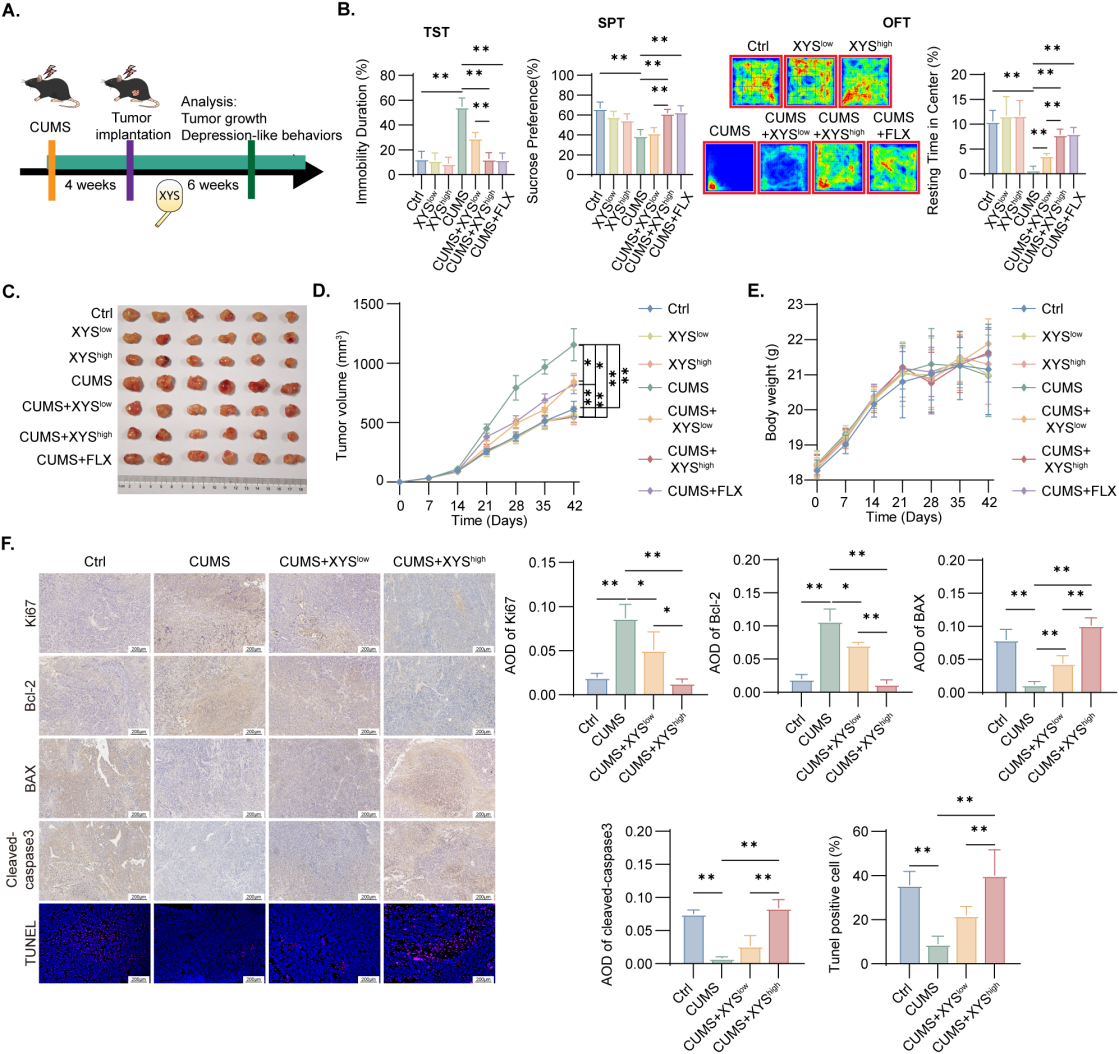
Effects of XYS on ED-TNBC mice. **(A)** Schematic of the CUMS model experiment. **(B)** Behavioral analysis of TST, SPT and OFT (n=6/group). **(C)** Representative Tumor images from mice (n=6/group). **(D)** Tumor growth curve in different groups (n=6/group). **(E)** Body weight change in each group (n=6/group). **(F)** Representative staining including Ki67, Bcl-2, BAX, cleaved-caspase3 and TUNEL in tumor tissues of different groups (n=6/group). Data are represented as mean ± SD, *P < 0.05, **P < 0.01.

To assess the therapeutic influence of XYS on ED-TNBC, a CUMS-induced TNBC mouse model was established using female C57BL/6 mice (**Figure 1A**). After four weeks of CUMS exposure, 1×10^6^ Py230 TNBC cells were orthotopically injected into the fourth mammary fat pad, followed by corresponding treatments. Compared with control group, CUMS exposure resulted in prolonged immobility time, reduced sucrose preference, and diminished locomotor activity, confirming the successful induction of depression-like behavior (**Figure 1B**). XYS administration produced dose-dependent behavioral improvement, and the high-dose regimen achieved antidepressant efficacy comparable to that of FLX (**Figure 1B**). Correlation analyses revealed a significant negative association between sucrose preference or time spent in the central zone and tumor volume, whereas immobility time in the tail suspension test exhibited a positive correlation with tumor volume, indicating a close relationship between depressive behavior and tumor progression (**Supplementary Figure S1**). CUMS exposure markedly promoted neoplastic growth, while XYS treatment substantially suppressed ED-TNBC progression in a dose-dependent manner (**Figures 1C, D**). Notably, XYS exhibited minimal antitumor activity in non-stressed controls, indicating its selective efficacy under ED conditions (**Figures 1C, D**). No significant alterations in body weight were detected during the treatment period, suggesting favorable tolerability (**Figure 1E**). Immunohistochemical analysis showed that CUMS significantly elevated the expression of Ki67 and Bcl-2 while reducing BAX and Cleaved-caspase-3 levels. XYS administration effectively reversed these molecular alterations (**Figure 1F**). Collectively, the results indicate that XYS selectively suppresses TNBC growth associated with ED.

### 3.2 Transcriptomics effects of XYS intervention on tumor tissue in TNBC mice under CUMS

**Figure 2:**
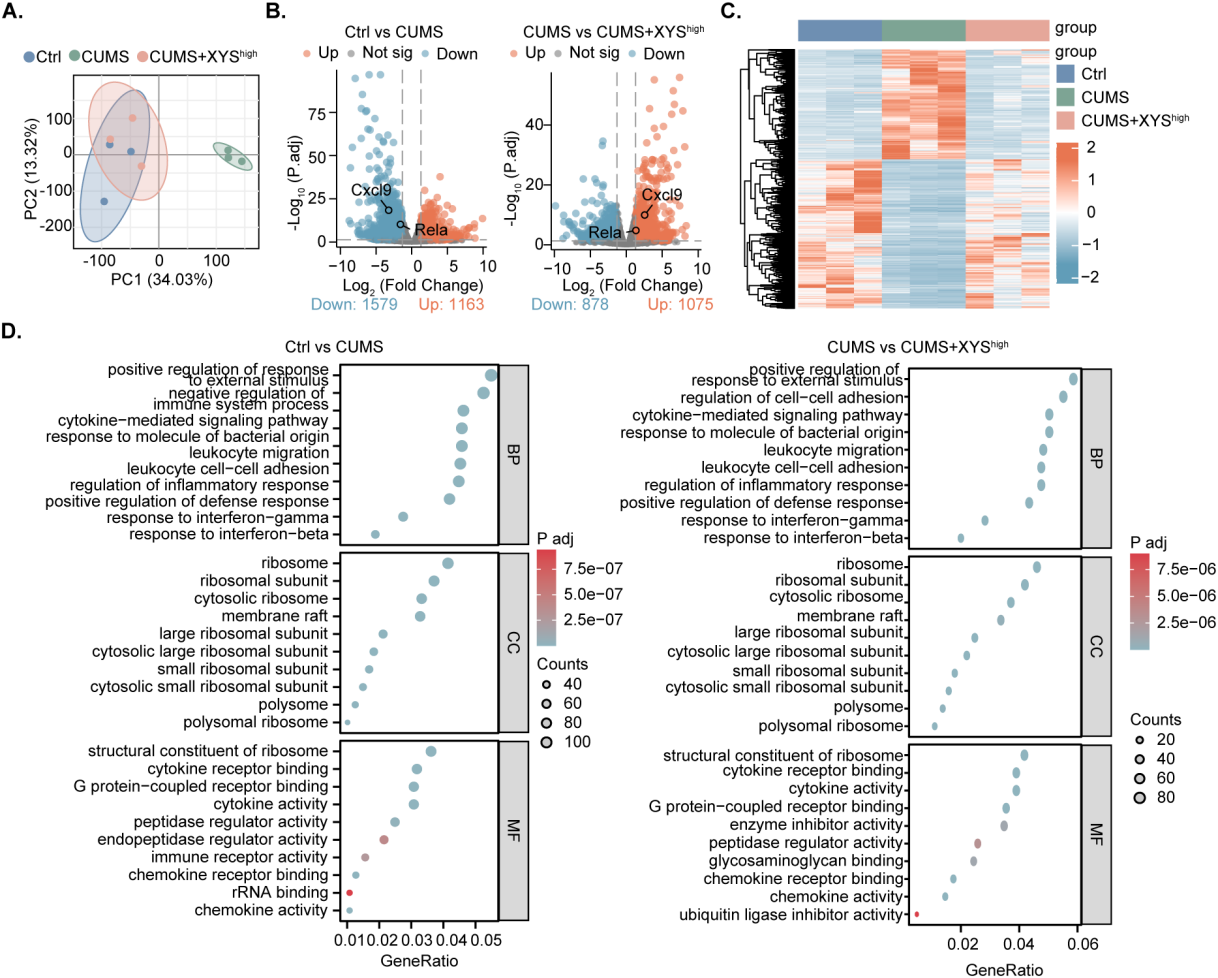
Transcriptomics changes in TNBC induce by CUMS after XYS intervention. **(A)** PCA analysis results of transcriptomes for the Ctrl, CUMS, and CUMS+XYS^high^ groups (n=3/group). **(B)** Volcano plot showing the number of upregulated and downregulated genes (Ctrl -vs- CUMS and CUMS -vs- CUMS+XYS^high^). **(C)** Heatmap of gene expression in each group (n=3/group). **(D)** GO enrichment analysis of differentially expressed genes (Ctrl -vs- CUMS and CUMS -vs- CUMS+XYS^high^).

To examine the regulatory effects of XYS on the TME in ED-TNBC, transcriptomic profiling (RNA-seq) was conducted using tumor tissues from the experimental mice. Principal component analysis (PCA) revealed clear segregation between the Ctrl and CUMS groups, while transcriptional profiles of the high-dose XYS group closely resembled those of the Ctrl group (**Figure 2A**). Differentially expressed genes (DEGs) were identified using thresholds of *P.adj* < *0.0*1 and |log_2_ fold change| > 1.3 (**Figures 2B, C**). Relative to the Ctrl and CUMS groups, 2742 DEGs were detected (1163 upregulated and 1579 downregulated), whereas comparison between CUMS and CUMS + XYS^high^ groups yielded 1953 DEGs (1075 upregulated and 878 downregulated). Gene Ontology (GO) enrichment analysis indicated that XYS modulated multiple immune-related processes, including regulation of the inflammatory response, activation of defense responses, and chemokine-mediated signaling (**Figure 2D**). *In vitro* assays demonstrated that TNBC cell proliferation remained unaffected by XYS-containing serum treatment (**Supplementary Figure S2**). These results indicate that XYS may attenuate TNBC progression under CUMS conditions primarily through modulation of the tumor immune microenvironment (TIME).

### 3.3 XYS enhanced the infiltration and function of CD8^+^T cell

**Figure 3:**
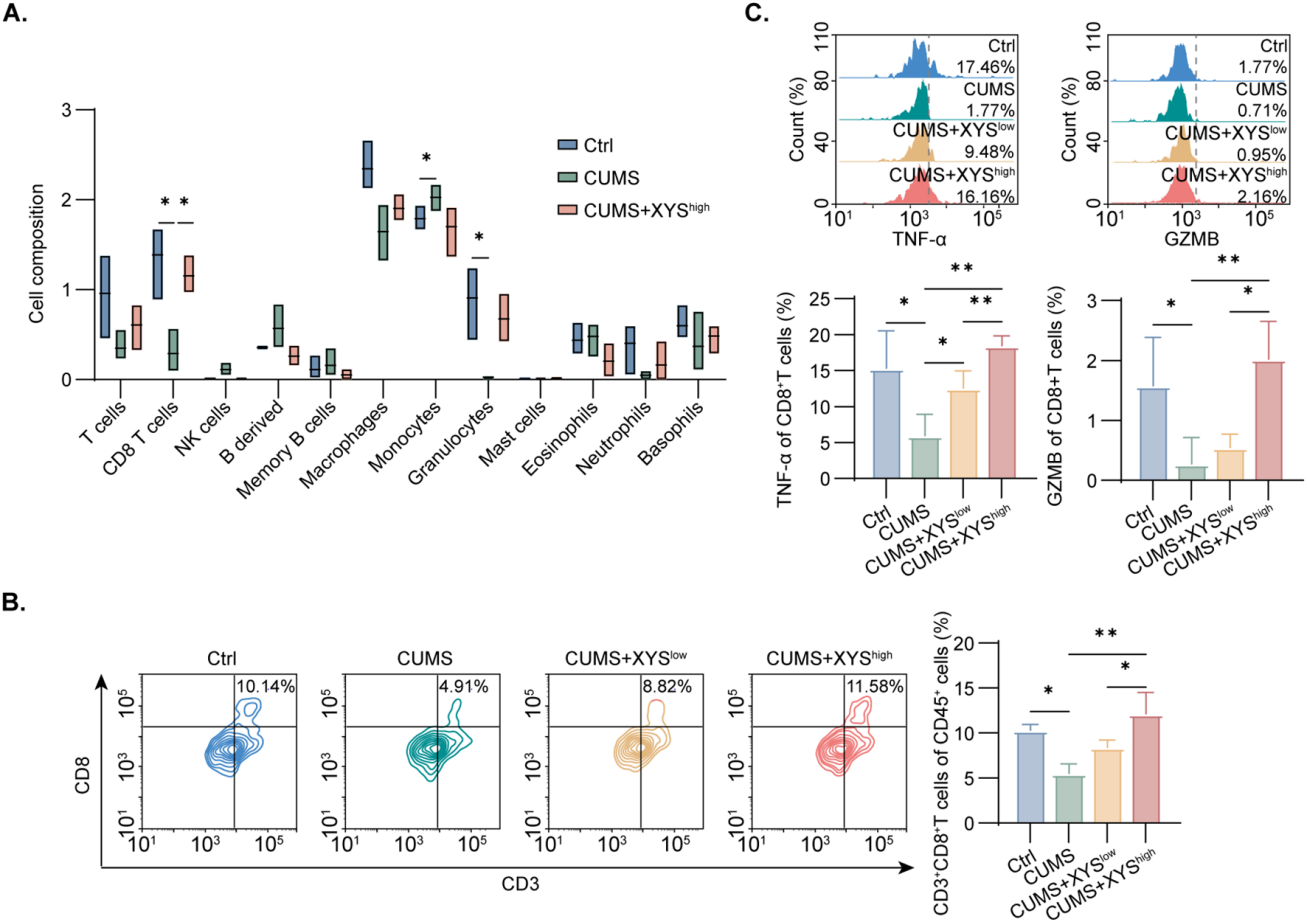
XYS increased CD8^+^T cell infiltration and function. **(A)** Immune infiltration analysis for the Ctrl, CUMS, and CUMS+XYS^high^ groups by immu_mMCPcounter (n=3/group). **(B)** Flow cytometry analysis of tumor-infiltrating CD8^+^T cell in each group (n=6/group). **(C)** Flow cytometry analysis of TNF-α and GZMB expression on CD8^+^T cell in each group (n=6/group). Data are represented as mean ± SD, *P < 0.05, **P < 0.01.

To further define the immunomodulatory mechanisms of XYS, immune cell composition was assessed using the immu_mMCPcounter algorithm. Analysis revealed significant reductions in CD8^+^ T cells and granulocytes, accompanied by increased monocyte infiltration in the CUMS group (**Figure 3A**). High-dose XYS treatment restored CD8^+^ T cell abundance to near-control levels (**Figure 3A**). Considering the essential role of CD8^+^ T cell as primary effector cells in antitumor immunity, subsequent analyses focused on this subset. Flow cytometry demonstrated that, compared to the CUMS group, high-dose XYS treatment substantially enhanced both the proportion and cytotoxic factors level (TNF-α and GZMB) of the intratumoral CD8^+^T cell (**Figures 3B, C**).

### 3.4 XYS promoted the CD8^+^T cell chemotaxis and cytotoxic activity by upregulating the Cxcl9

**Figure 4:**
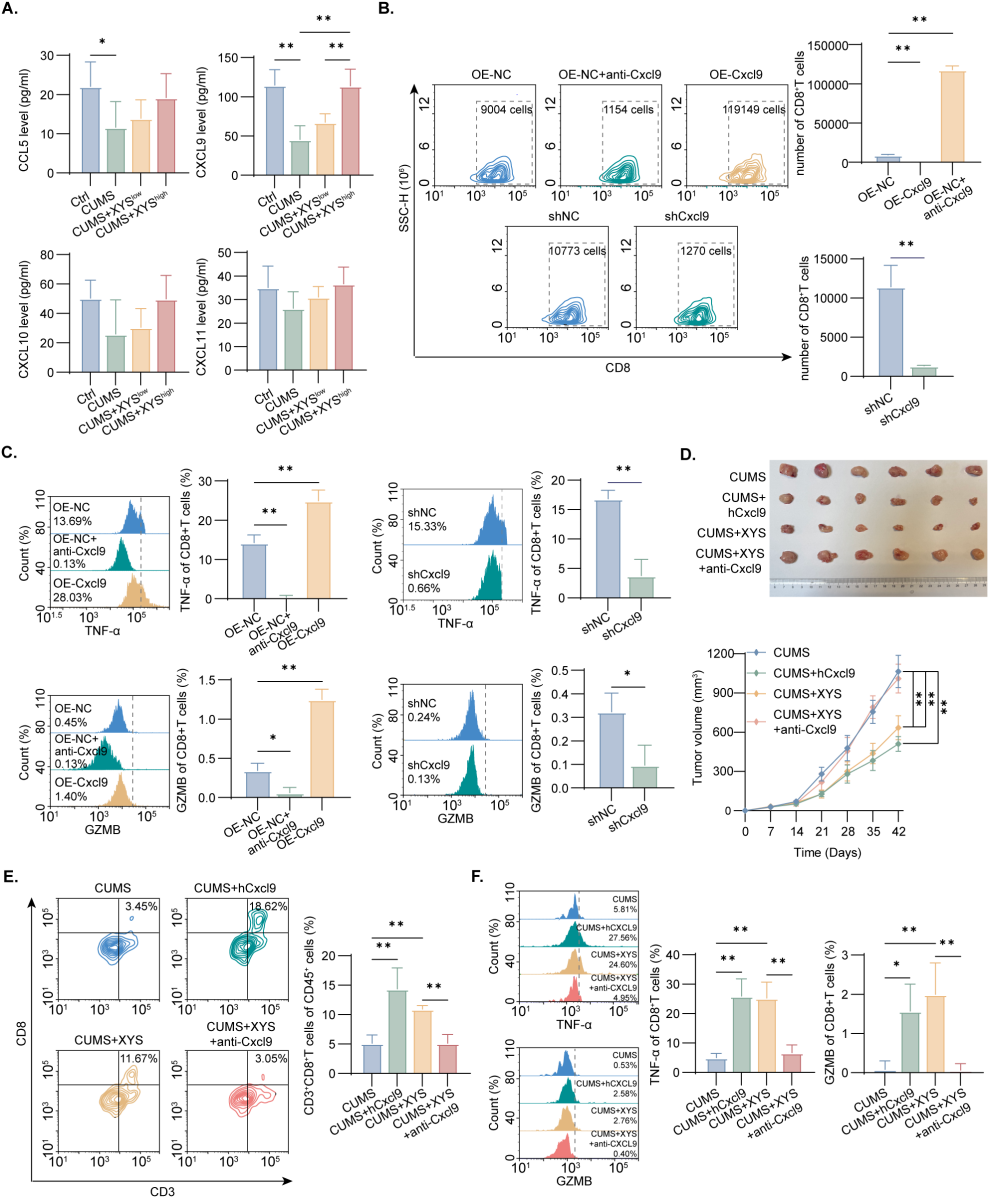
The effects of Cxcl9 in XYS inhibiting tumor growth and activating CD8^+^T cell under CUMS. **(A)** ELISA analysis of CD8^+^T cell related-chemokine expression in tumor tissues for the Ctrl, CUMS, CUMS+XYS^low^ and CUMS+XYS^high^ groups (n=6/group). **(B)** Quantitative analysis of CD8^+^T cell migration activity (n=3/group). **(C)** The quantification of TNF-α and GZMB rate in CD8^+^T cell (n=3/group). **(D)** Representative Tumor images and tumor growth curve in different groups (n=6/group). **(E)** Flow cytometry analysis of tumor-infiltrating CD8^+^T cell in each group (n=6/group). **(F)** Flow cytometry analysis of TNF-α and GZMB expression on CD8^+^T cell in each group (n=6/group). Data are represented as mean ± SD, *P < 0.05, **P < 0.01. OE-NC, overexpression negative control; OE-Cxcl9, overexpression of Cxcl9; shNC, knockdown negative control; shCxcl9, knockdown of Cxcl9.

Considering that CD8^+^ T cell infiltration is regulated by chemokines such as Ccl5 and Cxcl9–11 (35), chemokine levels in tumor tissues were quantified using ELISA. XYS administration markedly increased Cxcl9 protein expression in a dose-dependent manner in ED-TNBC (**Figure 4A**), and Cxcl9 levels exhibited strong positive correlations with both CD8^+^ T cell infiltration and activation markers (**Supplementary Figure S3**). qPCR analysis further confirmed a significant elevation of *Cxcl9* mRNA following high-dose XYS treatment (**Supplementary Figure S4**). In transwell assays, Cxcl9 overexpression in TNBC cells enhanced CD8^+^ T cell migration, whereas Cxcl9 knockdown or inhibition markedly reduced their chemotactic capacity (**Figure 4B**, **Supplementary Figures S5A, B**). Consistently, co-culture assays revealed that depletion or inhibition of Cxcl9 decreased TNF-α and GZMB secretion by CD8^+^ T cells, while Cxcl9 overexpression markedly increased their release (**Figure 4C**).

To verify the functional significance of Cxcl9 in mediating the *in vivo* effects of XYS, intratumoral Cxcl9 levels were manipulated through direct injection of Cxcl9-overexpressing Py230 cells or an anti-Cxcl9 monoclonal antibody. Injections were initiated two days prior to XYS administration to ensure effective modulation. Overexpression of Cxcl9 attenuated the tumor-promoting impact of CUMS, enhanced CD8^+^ T cell infiltration, and increased TNF-α and GZMB expression within the TME (**Figures 4D–F**; **Supplementary Figure S6**). In contrast, neutralization of Cxcl9 completely abolished the antitumor and CD8^+^ T cell–enhancing effects of XYS (**Figures 4D–F**; **Supplementary Figure S6**). Collectively, these results indicate that activation of the Cxcl9 signaling axis is indispensable for the therapeutic efficacy of XYS against ED-associated TNBC.

### 3.5 Chemical composition analysis and targets screening of XYS

**Figure 5:**
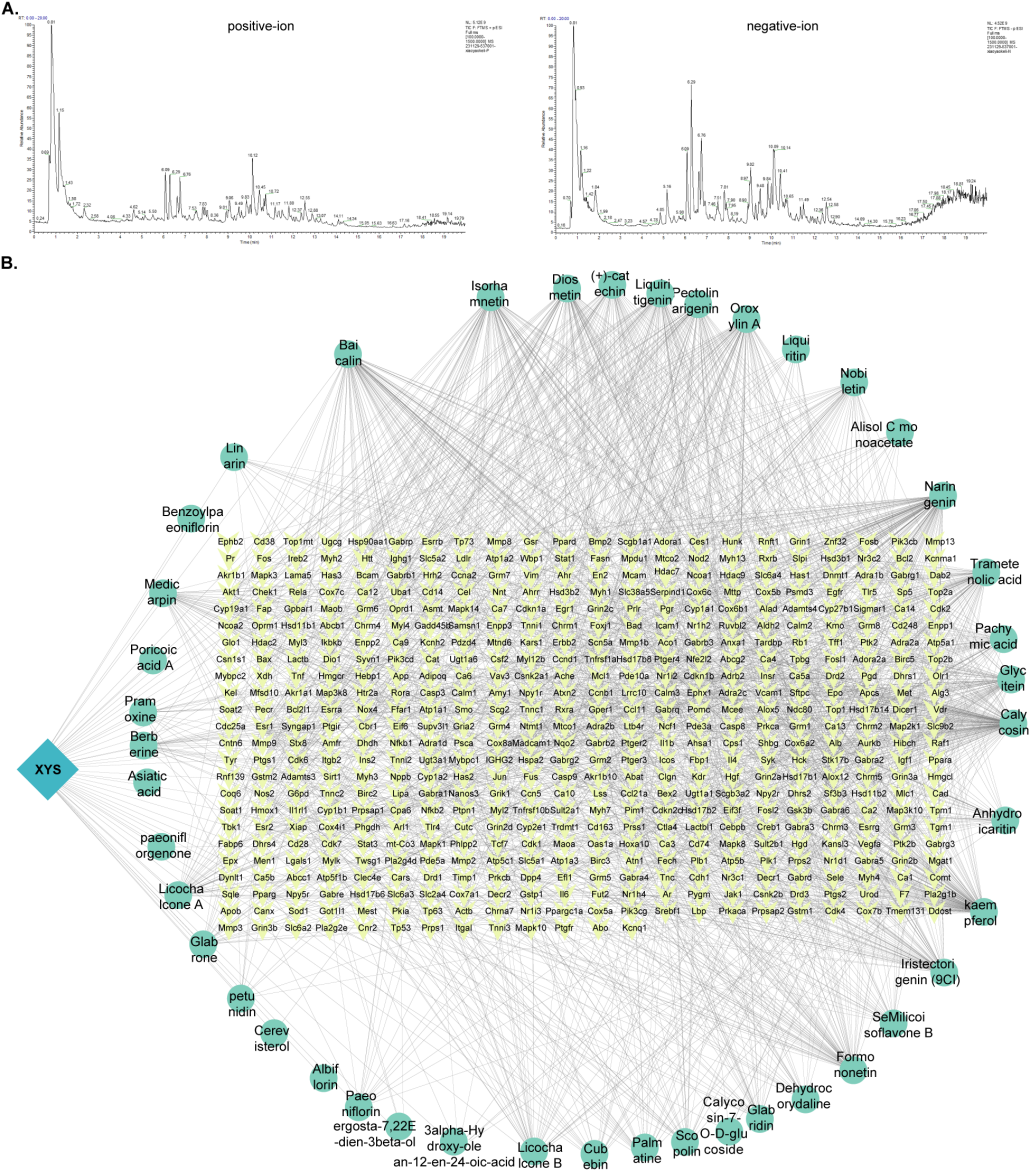
The major chemical constituents and targets of XYS. **(A)** Total ion chromatogram of XYS detected by UPLC-Q-Orbitrap-MS. **(B)** PPI network of the major bioactive constituents and their molecular targets.

Although the stimulation of Cxcl9 by XYS has been confirmed, the direct pathway by which XYS affects Cxcl9 secretion in ED-TNBC remains unclear. To delineate the chemical constituents of XYS, UPLC-Q-Orbitrap-MS analysis was conducted. Comparison of retention times, accurate masses, and molecular formulas with authentic standards and literature references enabled the identification of 351 distinct compounds (**Figure 5A**; **Supplementary Table S1**). Screening for pharmacologically relevant components based on oral bioavailability (OB > 30%) and drug-likeness (DL > 0.18) criteria yielded 43 bioactive constituents associated with 547 potential targets, as retrieved from authoritative TCM databases including TCMSP, TCMID, and TCM_suite (**Supplementary Table S2**). All targets were standardized to official mouse gene symbols using the UniProt database (**Supplementary Table S3**). A protein–protein interaction (PPI) network integrating the 43 active compounds and 520 validated targets was subsequently constructed to elucidate the potential molecular interactions underlying the therapeutic action of XYS (**Figure 5B**).

### 3.6 XYS targets the transcription factor Rela associated to Cxcl9

**Figure 6:**
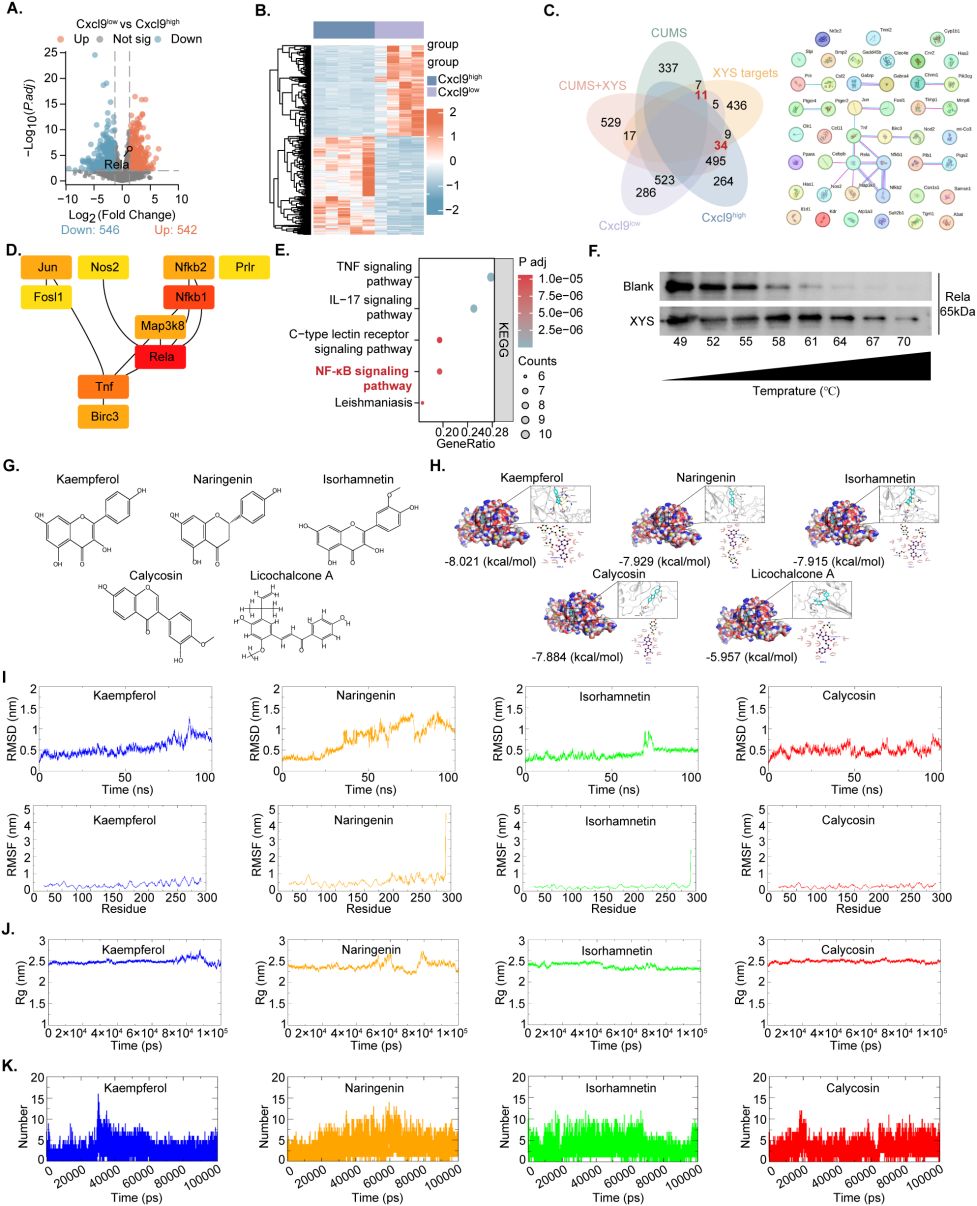
XYS targets the transcription factor Rela. **(A, B)** Volcano plot and heatmap showing the number of upregulated and downregulated genes (*Cxcl9^high^* -vs- *Cxcl9^low^*). **(C)** The XYS-*Cxcl9*-TNBC under CUMS targets are displayed in a venn diagram and PPI network diagram. **(D)** The top ten hub gene network of XYS to activate *Cxcl9* in TNBC under CUMS by the MCC algorithm. **(E)** The top five KEGG signaling pathways for the targets of XYS-*Cxcl9*-TNBC under CUMS. **(F)** CETSA analysis of XYS-Rela interactions in Py230. **(G)** Chemical structures of the major bioactive compounds in XYS that specifically target Rela. **(H)** Molecular docking of Rela with Nkaempferol, Naringenin, isorhamnetin, Calycosin and Licochalcone A. **(I)** RMSD and RMSF trajectory of molecular dynamics simulation for Rela with kaempferol, Naringenin, isorhamnetin and Calycosin. **(J)** Rg of kaempferol, Naringenin, isorhamnetin and Calycosin with Rela. **(K)** Hydrogen bond variations in molecular dynamics simulation for Rela with kaempferol, Naringenin, isorhamnetin and Calycosin.

To elucidate the molecular targets through which XYS enhances Cxcl9 secretion in ED-TNBC, potential targets associated with Cxcl9 expression in TNBC were systematically screened. Using the median *FPKM* value of Cxcl9 as a threshold, TNBC mice were stratified into *Cxcl9^high^* and *Cxcl9^low^* groups. DEGs were identified based on *P.adj* < *0.0*5 and |FC| > 1.3 (**Figures 6A, B**). Comparison between the two groups yielded 1088 DEGs, including 542 upregulated and 546 downregulated genes. Venn diagram analysis revealed 45 intersecting genes shared among XYS targets, Cxcl9-associated genes, and those regulated by XYS in ED-TNBC, comprising 34 upregulated and 11 downregulated genes (**Figure 6C**). The top 10 hub proteins were identified according to MCC scores calculated using CytoHubba in Cytoscape, enabling the prediction of key molecular regulators. *Rela* emerged as a core target mediating XYS-induced activation of *Cxcl9* in ED-TNBC (**Figure 6D**). KEGG pathway enrichment further indicated significant involvement of NF-κB signaling in XYS–*Cxcl9*–ED-TNBC–related targets (**Figure 6E**), prompting subsequent analysis of Rela expression in TNBC. Rela (p65), a key subunit of the NF-κB transcriptional complex, serves as an essential regulator of canonical NF-κB pathway activation, particularly in immune modulation and chemokine transcription (15). The interaction between XYS and Rela was further substantiated through a CETSA assay (**Figure 6F**). To further clarify the regulatory interaction between XYS and the Rela/NF-κB pathway, compounds within XYS capable of binding Rela were identified through reanalysis of the TCMSP, TCMID, and TCM_suite databases. The candidate molecules included Kaempferol, Naringenin, Isorhamnetin, Calycosin, and Licochalcone A (**Figure 6G**). Furthermore, we conducted molecular docking and dynamic studies to predict the probability and stability of effects that the active components of XYS may have on Rela. The results indicated that the active components of XYS tended to bind to the Rela protein (**Figure 6H**), with a binding affinity of -8.021 (kcal/mol) for Kaempferol, -7.929 (kcal/mol) for Naringenin, -7.915 (kcal/mol) for Isorhamnetin, -7.884 (kcal/mol) for Calycosin and -5.957 (kcal/mol) for Licochalcone A. Molecular dynamics analyses further confirmed the structural stability of these ligand–protein complexes (**Figures 6I–K**), indicating that XYS components may modulate Rela activity through direct interaction. Collectively, these results suggest that Rela serves as a potential molecular target mediating the regulatory effects of XYS.

### 3.7 XYS-mediated modulation of Cxcl9 expression was linked to Rela/NF-κB pathway activation

**Figure 7:**
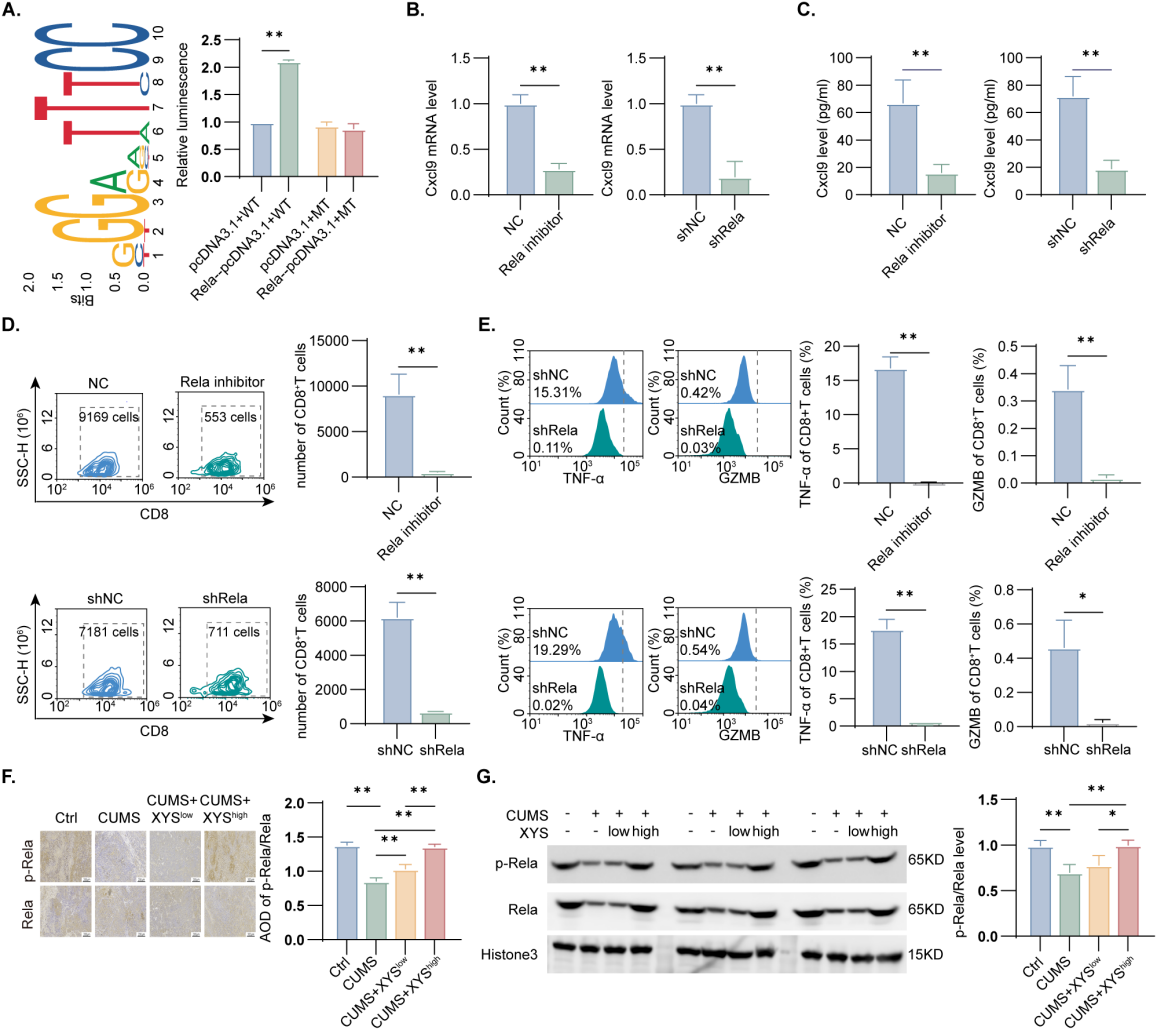
XYS improve the expression of Cxcl9 in TNBC under CUMS by modulating the Rela/NF-kB pathway. **(A)** The effect of *Rela* on *Cxcl9* promoter activity was examined by performing a dual luciferase reporter assay. **(B)** qPCR analysis of *Cxcl9* mRNA level of Py230 cells in each group (n=3/group). **(C)** ELISA analysis of Cxcl9 expression in Py230 after Rela modulating (n=3/group). **(D)** CD8^+^ T cell chemotaxis assays (n=3/group). **(E)** The quantification of TNF-α and GZMB rate in CD8^+^T cell was measured and compared (n=3/group). **(F)** Immunohistochemical staining of Rela and p-Rela in each group (n=6/group). **(G)** WB assays of Rela and p-Rela in each group (n=3/group). Data are represented as mean ± SD, *P < 0.05, **P < 0.01. OE-Rela, overexpression of Rela; shRela, knockdown of Rela.

Given the specific influence of XYS on Rela, the functional relevance of this interaction in mediating Cxcl9 upregulation was further examined. Luciferase reporter assays demonstrated that *Rela* markedly increased the transcriptional activity of the *Cxcl9* promoter region (P < 0.05, **Figure 7A**). Rela expression in Py230 cells was subsequently modulated through overexpression, knockdown, and pharmacological inhibition, with transfection efficiency of both *Rela*-*OE* and *shRela* constructs effectively validated (**FiguresS5C, D**). Alteration of *Rela* expression did not significantly affect TNBC cell proliferation (**Supplementary Figure S7**). However, *Rela* overexpression markedly elevated Cxcl9 expression (P < 0.05, **Supplementary Figures S8A, B**) and enhanced the capacity of TNBC cells to promote CD8^+^ T cell migration and activation (P < 0.05, **Supplementary Figures S8C-E**). In contrast, Rela silencing or inhibition significantly decreased Cxcl9 expression at both mRNA and protein levels (P < 0.01, **Figures 7B, C**) and suppressed CD8^+^ T cell migration as well as TNF-α and GZMB secretion (P < 0.05, **Figures 7D, E**). *In vivo* analysis further demonstrated that CUMS exposure reduced Rela nuclear translocation and phosphorylation, while high-dose XYS treatment effectively restored both processes (P < 0.01, **Figures 7F, G**). Collectively, these data indicate that activation of the Rela/NF-κB signaling axis mediates XYS-induced Cxcl9 upregulation.

## 4 Discussion

TNBC represents a highly heterogeneous subtype of breast cancer that continues to pose a substantial clinical challenge, characterized by poor prognosis and the lack of well-defined therapeutic targets (36). Accumulating evidence indicates that ED, including depression, not only accelerates TNBC progression but also constitutes an independent risk factor adversely affecting overall survival (37, 38). Retrospective studies have further shown that depression scores in TNBC patients are approximately 1.4 times higher than those observed in other BC subtypes (39). These data highlight the necessity for therapeutic strategies that transcend tumor-focused interventions to integrate psychological and emotional management in TNBC care. The mechanisms underlying ED-induced TNBC malignancy are multifaceted. Current medical approaches predominantly rely on Western pharmacotherapy, which, despite demonstrating partial efficacy, is frequently accompanied by substantial adverse effects that limit long-term clinical utility (40–43). In recent years, TCM has gained recognition as a potential therapeutic approach for managing malignancies associated with ED. Clinical observations indicate that XYS alleviates depressive symptoms in BC patients while enhancing treatment efficacy (20, 44). Despite these advances, the molecular mechanisms underlying the therapeutic actions of XYS remain unclear. In this study, a mouse model of ED-TNBC was developed using CUMS, followed by a comprehensive analytical strategy integrating transcriptomics, UPLC-Q-Orbitrap-MS, network pharmacology, molecular docking, and molecular dynamics simulations. This integrated approach aimed to identify the major bioactive components of XYS and to elucidate its molecular targets and mechanisms relevant to ED-TNBC therapy. The predicted mechanisms were further validated through a series of targeted *in vivo* and *in vitro* experiments.

In this study, XYS exhibited potent and selective antitumor activity against ED-TNBC. Transcriptomic profiling indicated that immune activation represented the principal feature of XYS-mediated remodeling of the TME in ED-TNBC. This observation holds particular relevance, as an immunologically “cold” TME constitutes a defining characteristic of cancer initiation and progression under ED conditions (4, 45). Immunologically “cold” tumor microenvironments typically exhibit reduced CD8^+^T cell infiltration - a key determinant of antitumor immunity (46). Previous research by Ye et al. (47) identified insufficient CD8^+^ T cell infiltration as a major factor contributing to depression-induced immunosuppression and accelerated tumor growth. The current results demonstrate that XYS treatment effectively reverses this immunosuppressive state by enhancing CD8^+^ T cell infiltration and augmenting the secretion of cytotoxic mediators, including TNF-α and GZMB, thereby converting a “cold” TME into an immunologically “hot” phenotype. This mechanism aligns with clinical observations that XYS improves antitumor immune responses in BC patients (20). Collectively, these results define a mechanistic association between the therapeutic efficacy of XYS in ED-TNBC and its capacity to restore CD8^+^ T cell–driven antitumor immunity. Besides, effective CD8^+^ T cell infiltration and functional activation are key determinants of responsiveness to immune checkpoint blockade, particularly in immunologically “cold” tumors. The ability of XYS to enhance CD8^+^ T cell recruitment and effector function suggests a potential role as an immunomodulatory adjunct to immune checkpoint blockers (ICBs) in ED-TNBC. Future studies could further elucidate the synergistic potential and mechanistic basis by conducting combination experiments with XYS and ICBs, integrated with dynamic monitoring of checkpoint molecule expression on both tumor and immune cells.

The indispensable antitumor functions of CD8^+^ T cells motivated an in-depth examination of the immunomodulatory mechanisms underlying XYS activity. Chemokine signaling within the TME plays a central role in regulating immune cell recruitment and activation, thereby influencing the transition of tumors from immunologically “cold” to “hot” phenotypes (48). The present analysis identified Cxcl9 as a key mediator responsible for promoting CD8^+^ T cell infiltration and cytotoxic activity in ED-TNBC. Previous studies have reported that Cxcl9 can be secreted by tumor cells (49), and tumor-derived Cxcl9 has been shown to attract CD8^+^ T cells to the peritumoral region and stimulate their cytolytic activity (15, 50, 51). These observations indicate that enhancing intratumoral Cxcl9 expression may represent an effective strategy for mitigating ED-TNBC progression. Nonetheless, the development of agents directly targeting Cxcl9 remains limited, and no clinically approved therapeutics modulating this pathway are currently available. Experimental evidence demonstrated that XYS treatment significantly increased Cxcl9 expression in ED-TNBC, whereas administration of neutralizing anti-Cxcl9 antibodies markedly reduced its therapeutic efficacy on CD8^+^ T cell infiltration and tumor suppression. Collectively, these data indicate that XYS inhibits tumor progression through activation of Cxcl9-dependent CD8^+^ T cell recruitment and antitumor immune responses, positioning XYS as a potential Cxcl9 inducer with clinical applicability in TIME modulation.

UPLC-Q-Orbitrap-MS analysis combined with network pharmacology was used to characterize the chemical constituents and potential targets of XYS. Cxcl9 was not directly identified as a target of XYS. Through hub target screening, molecular docking, and molecular dynamics simulation, Rela was identified as a key regulatory molecule mediating the effects of XYS in ED-TNBC. Rela is a central component of the cytokine-triggered NF-κB signaling cascade (52). Upon NF-κB pathway activation, Rela translocates into the nucleus to regulate gene transcription (52). Activation of the NF-κB signaling pathway has been recognized as an effective approach to modulating immune responses and enhancing antitumor immunity (15, 53). Studies using non-small cell lung cancer and melanoma models have demonstrated that NF-κB activation elevates Cxcl9 expression, thereby promoting CD8^+^ T cell tumor infiltration and antitumor activity (54–56). Experimental data indicated that knockdown or inhibition of Rela in TNBC cells suppressed Cxcl9 secretion and reduced the capacity of tumor cells to promote CD8^+^ T cell migration and cytotoxicity. Further analyses revealed that in ED-TNBC, XYS not only increased Rela mRNA and protein expression but also enhanced its nuclear translocation. Collectively, these results indicate that XYS-mediated regulation of Cxcl9 expression primarily depends on activation of the Rela/NF-κB signaling axis.

**Figure 8:**
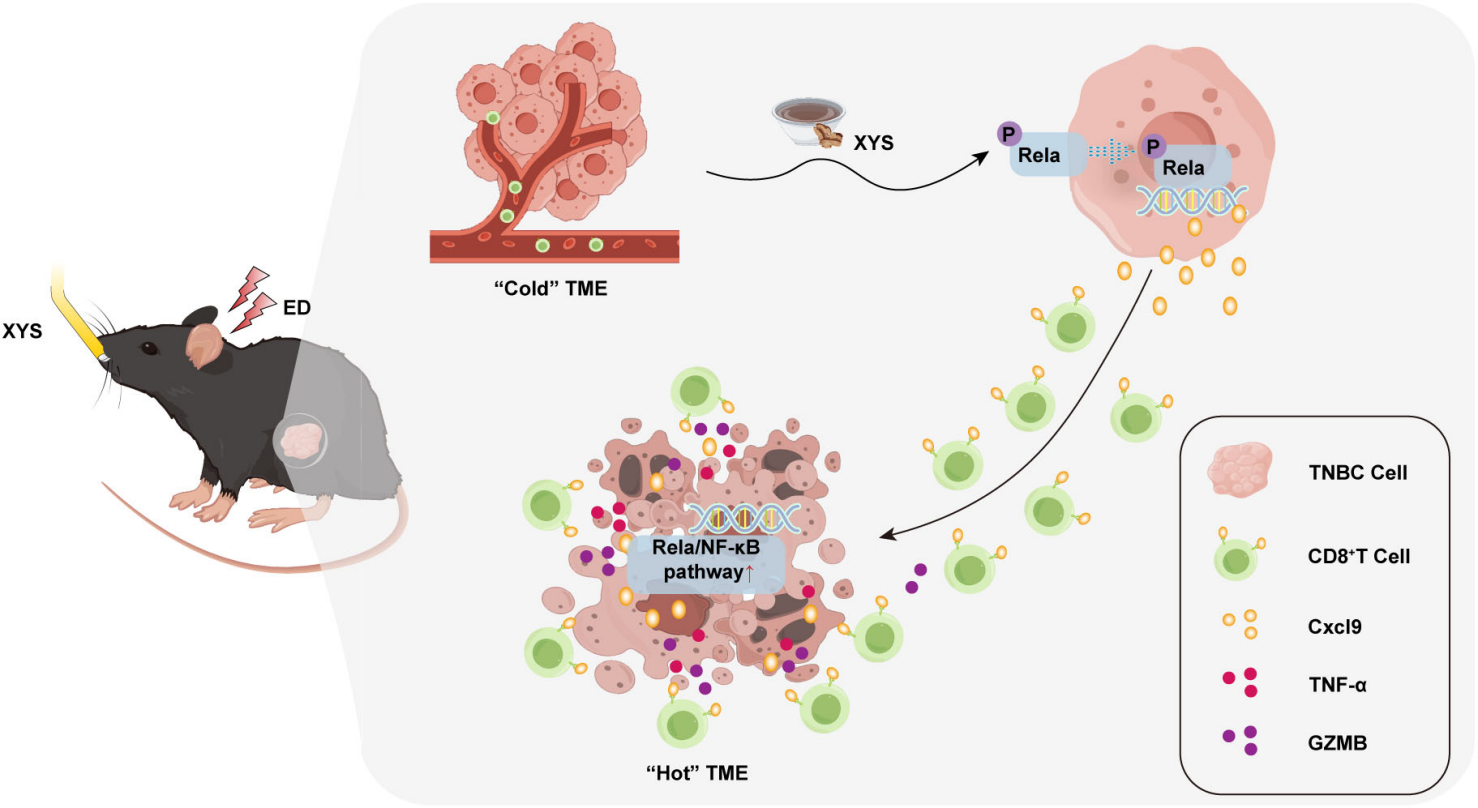
Molecular mechanisms of XYS in alleviates the growth of ED-TNBC.

The results indicate that ED markedly suppresses both the infiltration and cytotoxic activity of CD8^+^ T cells in TNBC, while XYS effectively reverses this immunosuppressive state through targeted activation of the Rela/NF-κB–Cxcl9 signaling axis (**Figure 8**). Nonetheless, several limitations should be noted. The present study relies on a single murine TNBC model (the Py230 cell line) and lacks validation in human clinical samples, which may limit the direct generalizability and translational applicability of the findings. Future investigations should incorporate validation in well-characterized patient cohorts and employ advanced mechanistic approaches, such as integrated immune profiling, to further elucidate the precise molecular interactions involved. Additionally, further studies dissecting the distinct roles of representative bioactive constituents may help refine the contribution of specific compounds and enhance the mechanistic resolution of the Rela/NF-kB-Cxcl9 axis.

## 5 Conclusion

The present study demonstrates that XYS attenuates ED-TNBC progression and counteracts ED-induced immunosuppression through modulation of the TIME. Specifically, XYS treatment in ED-TNBC (1) markedly increases Cxcl9 expression, thereby enhancing CD8^+^ T cell recruitment to tumor sites and promoting cytotoxic effector activity; and (2) activates the Rela/NF-κB signaling cascade, leading to the restoration of antitumor immune function. Collectively, these results provide a mechanistic basis for the application of XYS as an immunomodulatory therapeutic agent in ED-TNBC and highlight the therapeutic promise of targeting the Cxcl9 or Rela/NF-κB pathway to suppress ED-TNBC progression.

## Data Availability

The datasets presented in this study can be found in online repositories. The names of the repository/repositories and accession number(s) can be found in the article/**Supplementary Material**.

## Supplementary material

Supplementary Figure 1 Correlation analysis between behavioral parameters and tumor volume. *P < 0.05, **P < 0.01.

Supplementary Figure 2 Cell viability of Py230 Cells. Data are represented as mean ± SD.

Supplementary Figure 3 Correlation analysis between CD8^+^T cell related-chemokine expression and CD8^+^T cell. *P < 0.05, **P < 0.01.

Supplementary Figure 4 qPCR analysis of Cxcl9 mRNA level of tumor tissue in each group. Data are represented as mean ± SD, *P < 0.05, **P < 0.01.

Supplementary Figure 5 The Western blot images. **(A)** Validation of Cxcl9 overexpression. **(B)** Validation of Cxcl9 knockdown. **(C)** Validation of Rela overexpression. **(D)** Validation of Rela knockdown. **(E)** CETSA analysis of XYS-Rela interactions in Py230. **(F)** WB assays of Rela and p-Rela in each group.

Supplementary Figure 6 Representative staining including Ki67, Bcl-2, BAX, cleaved-caspase3 and TUNEL in tumor tissues of different groups. Data are represented as mean ± SD, *P < 0.05, **P < 0.01.

Supplementary Figure 7 Cell viability of Py230 Cells Under Rela Modulation. Data are represented as mean ± SD.

Supplementary Figure 8 Impact of Rela overexpression on Cxcl9 Expression and Chemotaxis, as well as functional activity of CD8^+^ T Cells. **(A)** qPCR analysis of *Cxcl9* mRNA level of Py230 cells in each group (n=3/group). **(B)** ELISA analysis of Cxcl9 expression in Py230 after Rela modulating (n=3/group). **(C)** CD8^+^ T cell chemotaxis assays (n=3/group). **(D, E)** The quantification of TNF-α and GZMB rate in CD8^+^T cell was measured and compared (n=3/group). Data are represented as mean ± SD, *P < 0.05, **P < 0.01.

Supplementary Table 1 The component of UPLC-Q-Orbitrap-MS analysis.

Supplementary Table 2 The major bioactive compounds and their corresponding targets.

Supplementary Table 3 The official gene symbols of mouse.
