# Supplementary figures and images for: Xiaoyao San alleviates emotional distress - induced TNBC growth through augmenting intratumoral CD8+T cell infiltration mediated by Rela/NF-kB-Cxcl9 axis
Source: Front Immunol. 2026 Feb 5;17:1762492. doi: 10.3389/fimmu.2026.1762492 (PMC12916637; doi:10.3389/fimmu.2026.1762492)

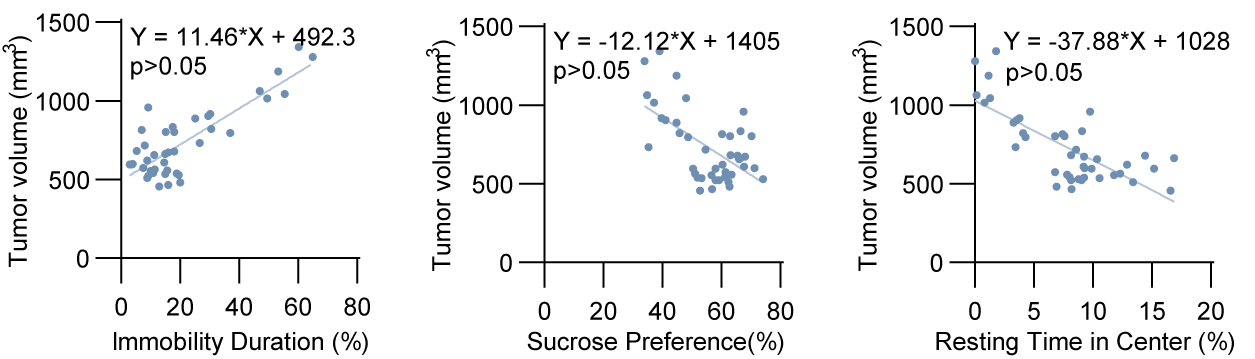

Supplement: Supplementary Figure 1 — Correlation analysis between behavioral parameters and tumor volume. *P < 0.05, **P < 0.01. [file Image1.tif]

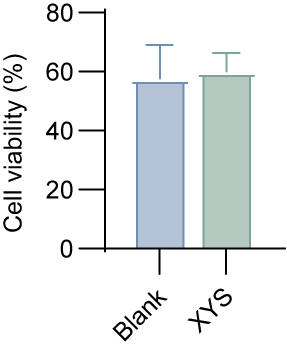

Supplement: Supplementary Figure 2 — Cell viability of Py230 Cells. Data are represented as mean ± SD. [file Image2.tif]

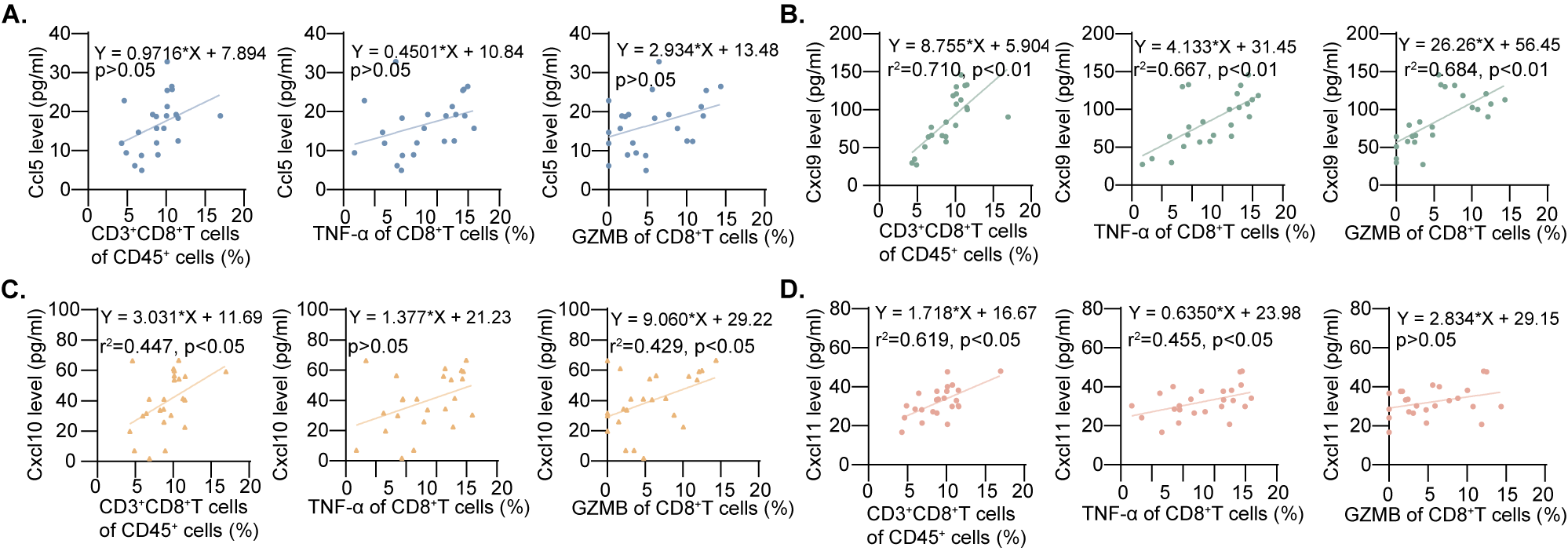

Supplement: Supplementary Figure 3 — Correlation analysis between CD8+T cell related-chemokine expression and CD8+T cell. *P < 0.05, **P < 0.01. [file Image3.tif]

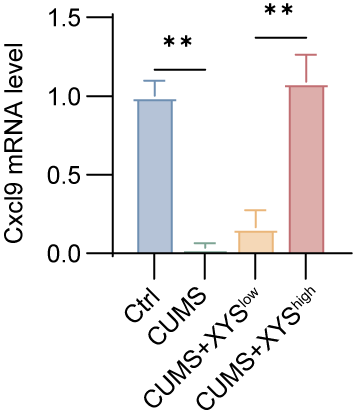

Supplement: Supplementary Figure 4 — qPCR analysis of Cxcl9 mRNA level of tumor tissue in each group. Data are represented as mean ± SD, *P < 0.05, **P < 0.01. [file Image4.tif]

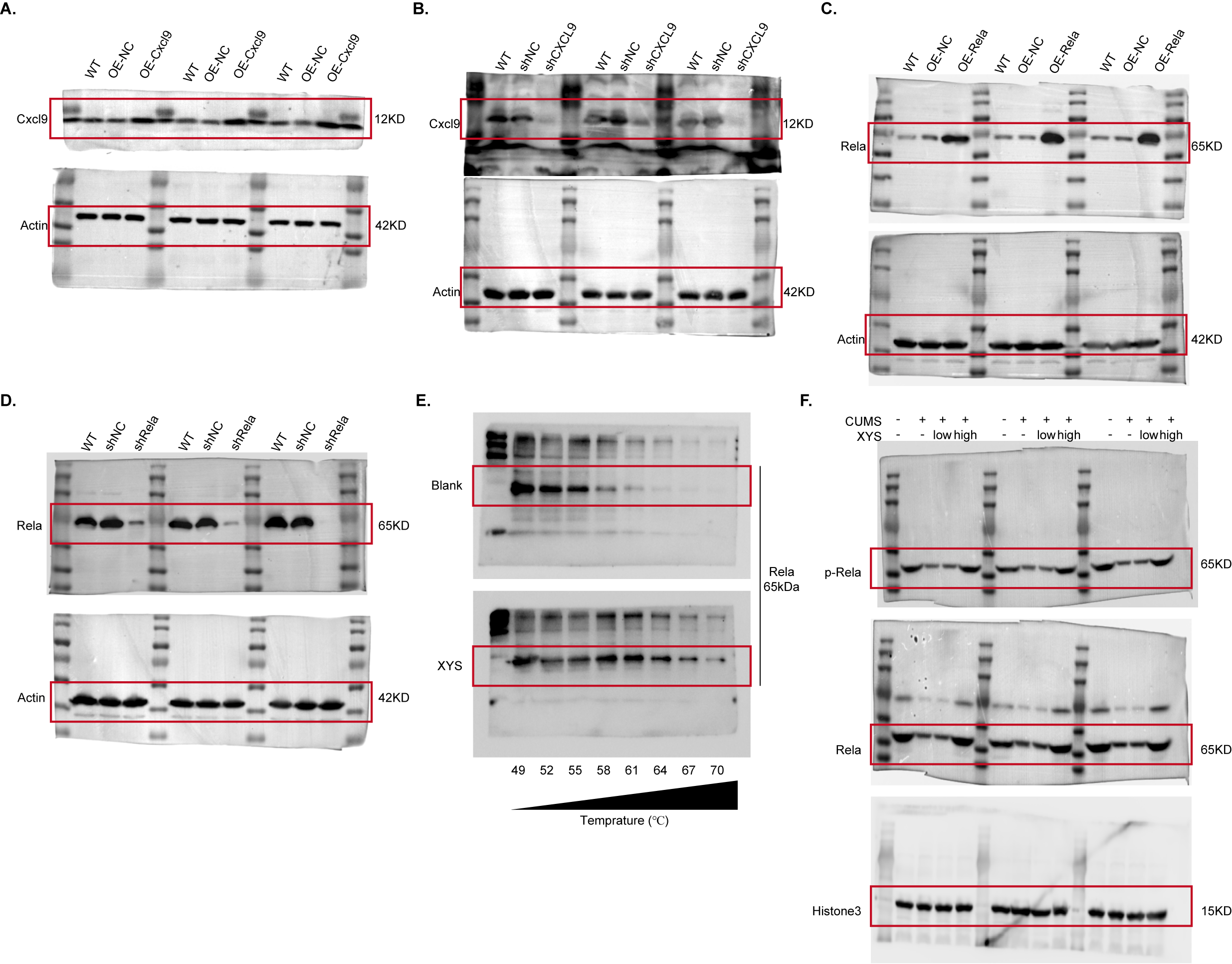

Supplement: Supplementary Figure 5 — The Western blot images. (A) Validation of Cxcl9 overexpression. (B) Validation of Cxcl9 knockdown. (C) Validation of Rela overexpression. (D) Validation of Rela knockdown. (E) CETSA analysis of XYS-Rela interactions in Py230. (F) WB assays of Rela and p-Rela in each group. [file Image5.tif]

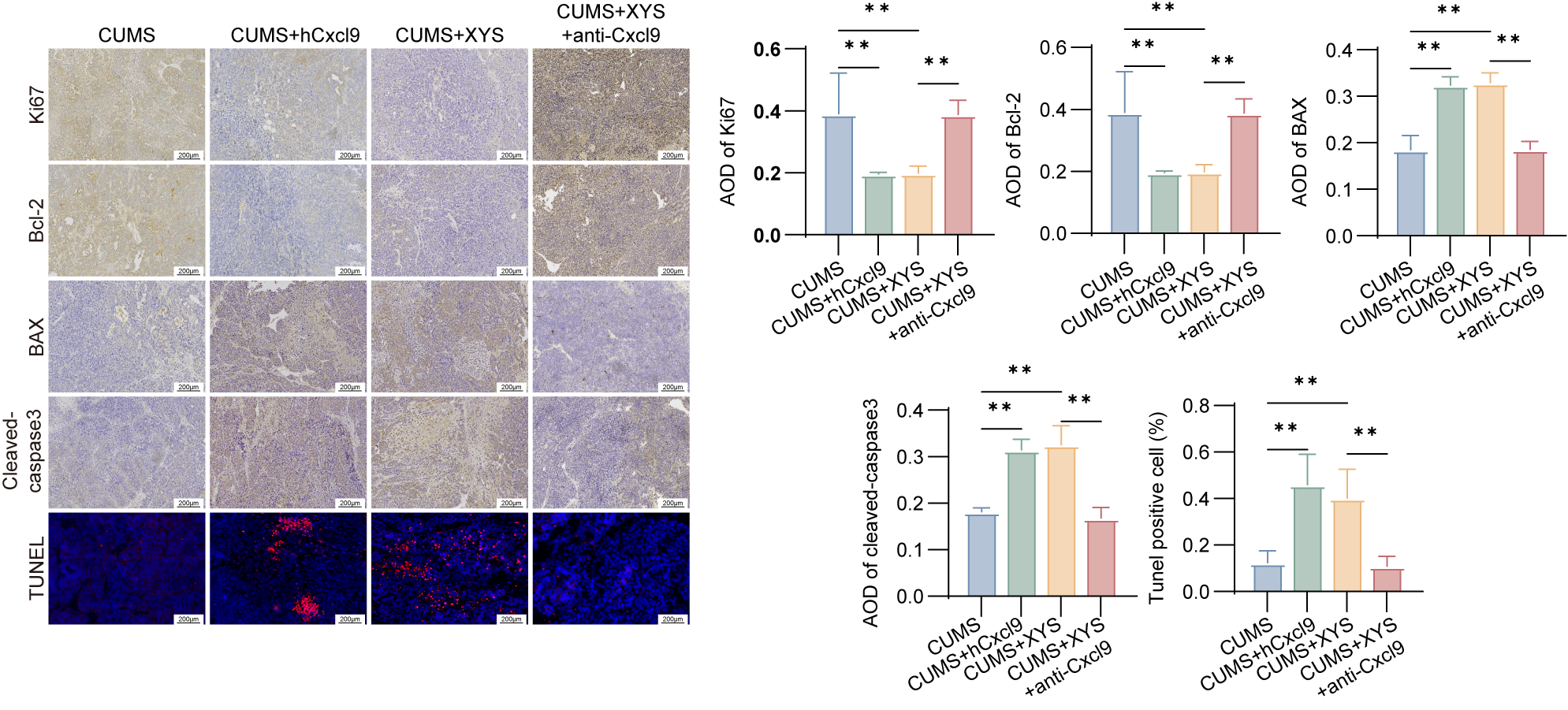

Supplement: Supplementary Figure 6 — Representative staining including Ki67, Bcl-2, BAX, cleaved-caspase3 and TUNEL in tumor tissues of different groups. Data are represented as mean ± SD, *P < 0.05, **P < 0.01. [file Image6.tif]

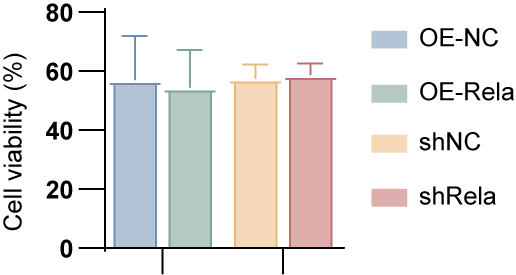

Supplement: Supplementary Figure 7 — Cell viability of Py230 Cells Under Rela Modulation. Data are represented as mean ± SD. [file Image7.tif]

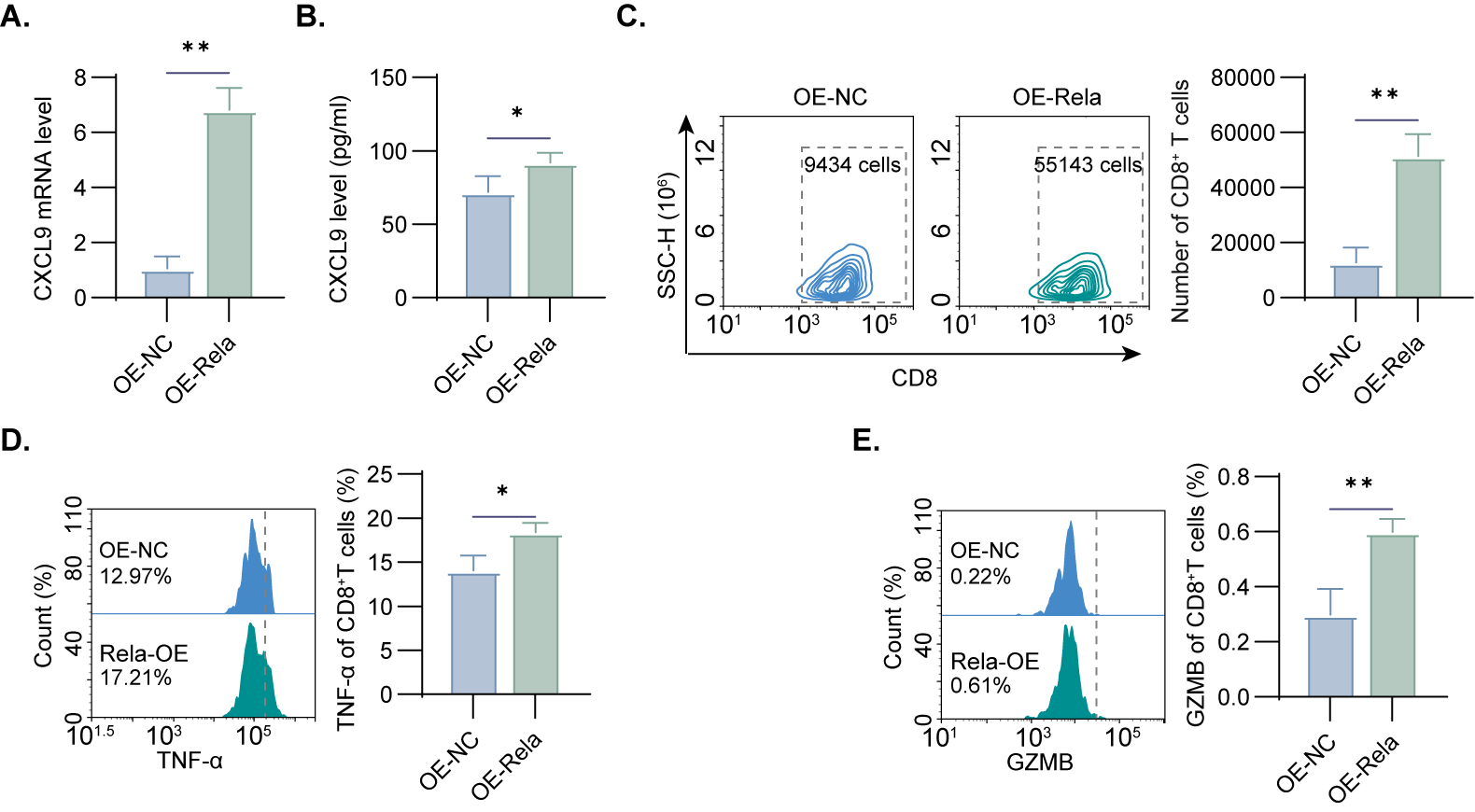

Supplement: Supplementary Figure 8 — Impact of Rela overexpression on Cxcl9 Expression and Chemotaxis, as well as functional activity of CD8+ T Cells. (A) qPCR analysis of Cxcl9 mRNA level of Py230 cells in each group (n=3/group). (B) ELISA analysis of Cxcl9 expression in Py230 after Rela modulating (n=3/group). (C) CD8+ T cell chemotaxis assays (n=3/group). (D, E) The quantification of TNF-α and GZMB rate in CD8+T cell was measured and compared (n=3/group). Data are represented as mean ± SD, *P < 0.05, **P < 0.01. [file Image8.tif]
